# Avicequinone B sensitizes anoikis in human lung cancer cells

**DOI:** 10.1186/s12929-018-0435-3

**Published:** 2018-04-09

**Authors:** Arisara Prateep, Somruethai Sumkhemthong, Wiranpat Karnsomwan, Wanchai De-Eknamkul, Supakarn Chamni, Pithi Chanvorachote, Chatchai Chaotham

**Affiliations:** 1 Department of Biochemistry and Microbiology, Faculty of Pharmaceutical Sciences, Chulalongkorn University, Bangkok, 10330 Thailand; 2 Department of Pharmacognosy and Pharmaceutical Botany, Faculty of Pharmaceutical Sciences, Chulalongkorn University, 10330 Bangkok, Thailand; 3 Department of Pharmacology and Physiology, Faculty of Pharmaceutical Sciences, Chulalongkorn University, Bangkok, 10330 Thailand; 4 Cell-based Drug and Health Products Development Research Unit, Faculty of Pharmaceutical Sciences, Chulalongkorn University, Bangkok, 10330 Thailand

**Keywords:** Lung cancer, Anoikis, Survival pathway, Avicequinone B, Furanonaphthoquinone

## Abstract

**Background:**

During metastasis, cancer cells require anokis resistant mechanism to survive until reach the distant secondary tissues. As anoikis sensitization may benefit for cancer therapy, this study demonstrated the potential of avicequinone B, a natural furanonaphthoquinone found in mangrove tree (Avicenniaceae) to sensitize anoikis in human lung cancer cells.

**Methods:**

Anoikis inducing effect was investigated in human lung cancer H460, H292 and H23 cells that were cultured in ultra-low attachment plate with non-cytotoxic concentrations of avicequinone B. Viability of detached cells was evaluated by XTT assay at 0–24 h of incubation time. Soft agar assay was performed to investigate the inhibitory effect of avicequinone B on anchorage-independent growth. The alteration of anoikis regulating molecules including survival and apoptosis proteins were elucidated by western blot analysis.

**Results:**

Avicequinone B at 4 μM significantly induced anoikis and inhibited proliferation under detachment condition in various human lung cancer cells. The reduction of anti-apoptotic proteins including anti-apoptotic protein B-cell lymphoma 2 (Bcl-2) and myeloid cell leukemia 1 (Mcl-1) associating with the diminution of integrin/focal adhesion kinase (FAK)/Proto-oncogene tyrosine-protein kinase (Src) signals were detected in avicequinone B-treated cells.

**Conclusions:**

Avicequinone B sensitized anoikis in human lung cancer cells through down-regulation of anti-apoptosis proteins and integrin-mediated survival signaling.

## Background

Lung cancer is a high prevalence and leading cause of death in cancer patients worldwide [1]. Metastasis or the spreading of cancer cells from primary site to secondary vital organs is a major cause of mortality in the lung cancer [2, 3]. According to highly metastatic features, most of lung cancer patients are frequently diagnosed at advanced stage presenting dissemination of tumor pathology [4, 5]. Such concepts lead to the urgent need for anti-metastasis therapy for lung cancer.

In the process of metastasis, most population of detached cancer cells should die by the mechanism of detachment-induced apoptosis termed “anoikis” [6]. This specific pattern of cell death is caused by the reduction of cellular survival signals providing by the adhesion of the cells to appropriate surface or membrane through integrins [7]. Loss of the interaction between integrins, cellular adhesive molecules and extracellular matrix (ECM) leads to the deprivation of survival signals following with apoptosis cell death in non-adherent cells [8].

Anoikis is believed to be a critical mechanism in preventing non-adherent cell growth and the growth of cells in an inappropriate environment [9]. In order to survive in blood or lymphatic circulation, certain cancer cells acquire anoikis resistance mechanisms. In metastatic cells, anoikis is inhibited by the increase of survival signals through the modulation of integrins expression contributing activation of FAK (focal adhesion kinase)/Src (proto-oncogene tyrosine-protein kinase) and PI3K (phosphatidylinositol-4,5-bisphosphate 3-kinase)/AKT (phospho kinase B) pathway [10–14]. Caveolin-1 was previously shown to play a role in attenuating anoikis response in lung cancer cells by maintain the level of Mcl-1 [15–17]. Moreover, the up-regulation of anti-apoptosis Bcl-2 family proteins including Bcl-2 (B-cell lymphoma 2) and Mcl-1 (myeloid cell leukemia 1) has been associated with anoikis resistance and highly metastasis cancer cells [18–20].

Avicequinone B, naphtho [2, 3-b] furan-4, 9-dione isolated from mangrove tree such as *Avicennia alba* and *Avicennia marina* has been shown to possess several pharmacological activities [21]. Anticancer activity of naphthoquinone derivatives have been illustrated through the induction of apoptosis and the inhibition on migration and invasion [22, 23]. So far, the potentials of these furanonaphthoquinone compounds for sensitizing anoikis and their regulatory approaches are largely unknown. We aimed to investigate the anoikis sensitizing effect and the underlying mechanisms of action of avicequinone B in human lung cancer cells. The information obtained from this study will emphasize the therapeutic benefits of avicequinone B for further development as an effective anticancer drug.

## Method

### Chemical reagents

All chemical reagents used for synthesis of avicequinone B and cell culture including XTT (2,3-b-(2-methoxy-4-nitro-5-sulfophenyl)-2H-tetrazolium-5-carboxanilide salt), MTT (3-(4,5-Dimethylthiazol-2-yl)-2,5-diphenyltetrazolium bromide), Hoechst33342, propidium iodide (PI), DMSO (dimethysulfoxide) and agarose were purchased from Sigma Chemical, Inc. (St. Louis, MO, USA). Annexin V-FITC for apoptosis detection was provided by Thermo Fisher Scientific (Waltham, MA, USA). Primary antibody of Bcl-2, Mcl-1, Bax (Bcl-2-associated X protein), caveolin-1, integrin β1, integrin β3, FAK, p-FAK (Try 397), Src, p-Src (Try 418), AKT, p-AKT (Ser 473), ERK (extracellular signal–regulated kinase), p-ERK (Thr 981), β-actin and specific horseradish peroxidase (HRP)-link secondary antibody were obtained from Cell Signaling Technology, Inc. (Danver, MA, USA). Supersignal West Pico, a chemiluminescence substrate for western blot analysis was purchased from Thermo Fisher Scientific (Waltham, MA, USA). Protease inhibitor cocktail and Bicinchoninic acid (BCA) protein assay kit were obtained from Roche Applied Science (Indianapolis, IN, USA) and Pierce Biotechnology (Rockford, IL, USA), respectively.

### Preparation of avicequinone B

**Fig. 1:**
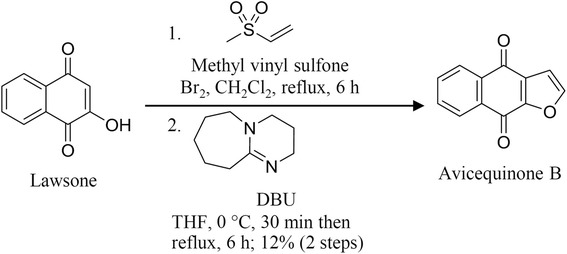
Synthetic scheme of avicequinone B

Avicequinone B was prepared from chemical synthesis using a facile synthesis as previous report [24]. Briefly, anhydrous solvents were dried over 4 Å molecular sieves. Methyl vinyl sulfone (4.71 mmol, 500 mg) was dissolved in dry dichloromethane (CH_2_Cl_2_, 10 ml) in a 50-mL oven-dried round-bottomed flask. The reaction mixture was stirred at room temperature under an argon atmosphere. Next, neat bromine (Br_2_, 7.07 mmol, 0.2 ml) was slowly added into the reaction. Then, the reaction mixture was refluxed for 6 h, concentrated under reduce pressure and reconstituted in dry tetrahydrofuran (THF, 20 ml). The reaction solution was then cooled at 0 °C and 1,8-diazabicyclo[5.4.0]undec-7-ene (DBU, 7.07 mmol, 1.1 ml) was slowly added dropwise over 20 min. The reaction mixture was stirred at 0 °C for 30 min. Next, lawsone (4.71 mmol, 820.2 mg) was added and another portion of DBU (7.07 mmol, 1.1 ml) was slowly added dropwise over 20 min. The reaction mixture was stirred at 0 °C for 30 min. The reaction was warmed up to room temperature and heated to reflux for 6 h. The reaction was then concentrated under reduced pressure and the residue was dissolved in dichloromethane (100 ml), washed with water (100 ml) and saturated aqueous ammonium chloride (100 ml). The organic layer was separated and the aqueous layer was extracted with dichloromethane (50 ml × 3 times). The combined organic layer was dried over anhydrous sodium sulfate and concentrated to obtain the crude product. The crude product was purified over silica gel column chromatography using dichloromethane: hexanes (3:1 *v*/v) as eluent to provide avicequinone B as a pale yellow solid at a yield of 69 mg (12%). ^1^H-NMR (CDCl_3_, 300 MHz) δ 8.22 (1H, m, 5-H), 8.22 (1H, m, 8-H), 7.77 (1H, m, 6-H), 7.77 (1H, m, 7-H), 7.77 (1H, d, J = 1.5 Hz, 2-H), 7.01 (1H, d, J = 1.5 Hz 3-H); ^13^C-NMR (CDCl_3_, 300 MHz) δ 180.6 (C-4), 173.6 (C-9), 152.7 (C-9a), 148.6 (C-2), 132.5 (C-4a), 134.0 (C-7), 133.9 (C-6), 133.2 (C-8a), 130.5 (C-3a), 127.1 (C-8), 127.0 (C-5), 108.7 (C-3); IR (KBr) 3142, 2853, 1683, 1585, 1566, 1478, 1365, 1206, 1182, 952, 714 cm^− 1^; HRMS-ESI m/z 221.0212 ([M + Na]^+^, calcd for C_12_H_6_O_3_Na^+^ 221.0215). Spectroscopic data of avicequinone B were matched with the previous report. The synthetic scheme of avicequinone B was demonstrated in Fig. 1.

### Cell culture

Human lung cancer H460, H292 and H23 cells were obtained from ATCC, American Type Culture Collection (Manassas, VA, USA). They were maintained in RPMI (Roswell Park Memorial Institute) medium supplemented with 10% fetal bovine serum (FBS), 2 mmol/l l-glutamine and 100 units/ml penicillin/streptomycin (Gibco, Gaithersburg, MA, USA) under 5% CO_2_ at 37 °C. The cells that reached 70–80% confluence were used for next experiments.

### Cell viability assay

Cell viability of adherent cell was evaluated by MTT assay. Briefly, lung cancer cells were cultured in complete RPMI medium containing various concentrations (0–10 μM) of avicequinone B for 24 h. The cells were then incubated with 0.4 mg/ml of MTT solution at 37 °C in a dark place for 4 h. The MTT solution was replaced with DMSO (100 μl/well) to dissolve the purple formazan crystal. The intensity of formazan color was measured at 570 nm using a microplate reader (Anthros, Durham, NC, USA). Percentage of cell viability in relation to the non-treated control was calculated from the optical density (OD) ratio of treated to non-treated control cells.

In order to evaluate anti-proliferative activity of avicequinone B, human lung cancer H460 cells were prepared at density of 2 × 10^3^ cells/well in 96 well-plate. The cells were incubated with non-toxic concentrations of avicequinone B or left untreated for 24, 48 and 72 h then cell viability was examined via MTT assay. The %cell proliferation was obtained from the calculation between OD of the cells at each time point divided by OD of non-treated control at 24 h.

### Detachment-induced cell death

A single-cell suspension of human lung cancer cells at a density of 1.5 × 10^5^ cells/ml was prepared in RPMI culture medium. In order to prevent anchorage, lung cancer single-cell suspension was culture in an ultra-low attachment plate (Corning, Acton, MA, USA). The cells were treated with non-toxic concentrations of avicequinone B for 0, 6, 9, 12 and 24 h or left untreated. After indicated time point, the cells were harvested for evaluation of cell viability through the incubation with 20 μM of XTT at 37 °C for 4 h. The intensity of the formazan product from XTT was measured at 450 nm using a microplate reader.

### Detection of mode of cell death

Nuclear staining with Hoechst33342 (10 μM) and PI (5 μg/ml) was performed to detect apoptosis and necrosis cell death in human lung cancer cells treated with avicequinone B. Bright blue fluorescence of Hoechst33342 and red fluorescence of PI were observed and evaluated under fluorescent microscope (Olympus IX51 with DP70; Olympus, Melville, NY, USA) as apoptosis and necrosis cells, respectively. For evaluation on detachment-induced apoptosis or anoikis, single-cell suspension of human lung cancer cells treated non-toxic concentrations of avicequinone B for 24 h in ultra-low attachment plate were collected and co-stained with Hoechst33342/PI [25, 26]. Mode of cell death were examined and counted under fluorescent microscope.

The relative ratio of amount of apoptosis cells to total cell number was presented as %apoptosis and %anoikis for attachment and detachment culture condition, respectively.

### Annexin V/PI flow cytometry analysis

Anoikis in non-adherent cells was further evaluated through flow cytometry using an Annexin V-FITC apoptosis assay kit. Single-cell suspension of H460 cells cultured in an ultra-low attachment 6 well-plate at a density of 1.5 × 10^5^ cells/ml were collected after incubated with 0–4 μM of avicequinone B for 24 h. The cells were washed and resuspended in phosphate buffer saline (PBS), pH 7.4. The single-cell suspensions were then centrifuged and dispersed in binding buffer (100 μl). Annexin V-FITC (1 μg/ml) and PI (2.5 μg/ml) were added into the cell suspensions as recommended in the manufacturer’s instructions. Living, apoptosis and necrosis cells were analyzed via a FACScan flow cytometer using CellQuest software (Becton-Dickinson, Redlands, CA, USA).

### Anchorage-independent growth assay

The ability to proliferate under detachment condition of human lung cancer cells was investigated in soft agar assay. Each well of a 24 well-plate was covered with 500 μl of 0.5% agarose in complete RPMI medium. In order to prevent spontaneous aggregation of detached cells, the single-cell suspension was dispersed in 0.33% agarose in culture medium which is the optimum condition to prevent cell aggregation and permit cell growth [27]. The single-cell suspensions of lung cancer cells in 0.33% agarose (1500 cells/250 μl) with different concentrations of avicequinone B were prepared and placed on solidified 0.5% agarose gel. After the upper layer was left to set in the incubator at 37 °C for 4 h, 250 μl of culture medium was added on top and every 3 days. The formation of cancer colonies was investigated under a microscope (Olympus IX51 with DP70) after 14 days.

### Western blot analysis

The alteration of anoikis-regulating proteins in lung cancer cells was analyzed by western blot analysis. Single-cell suspensions of H460 cells at a density of 1.5 × 10^5^ cells/ml were incubated with avicequinone B (0–4 μM) for 12 h. Then, the cells were harvested and lysed with lysis buffer containing 20 mM Tris–HCl (pH 7.5), 1% Triton X-100, 150 mM sodium chloride, 10% glycerol, 1 mM sodium orthovanadate, 50 mM sodium fluoride, 100 mM phenylmethylsulfonyl fluoride, and a commercial protease inhibitor mixture (Roche Applied Science) at 4 °C for 60 min. Cell lysates were collected and determined for total protein content by using the BCA protein assay kit (Pierce, Rockford, IL, USA). An equal amount of protein from each sample was resolved under denaturing conditions by 10% SDS-PAGE and transferred onto a nitrocellulose membrane. The membranes are blocked for 1 h in 5% non-fat dry milk in TBST (25 mM Tris–HCl, pH 7.4, 125 mM sodium chloride, 0.05% Tween 20) before the incubation with specific primary antibody at 4 °C for 12 h. After washing three times (5 min) with TBST, the membranes were probed with HRP-conjugated secondary antibody for 2 h at room temperature. The signal of immunoreactive proteins was detected by enhanced chemiluminescence (Supersignal West Pico; Thermo Fisher Scientific, Waltham, MA, USA). The quantitative analysis was performed with the analyst/PC densitometry software (Bio-Rad Laboratories, Hercules, CA, USA).

### Statistical analysis

Mean data from three independent experiments were normalized to result of non-treated control. Statistical analysis was performed using one-way ANOVA following with post-hoc test. *p* < 0.05 was considered as statistically significant.

## Results

### Cytotoxicity of avicequinone B in human lung cancer cells

**Fig. 2:**
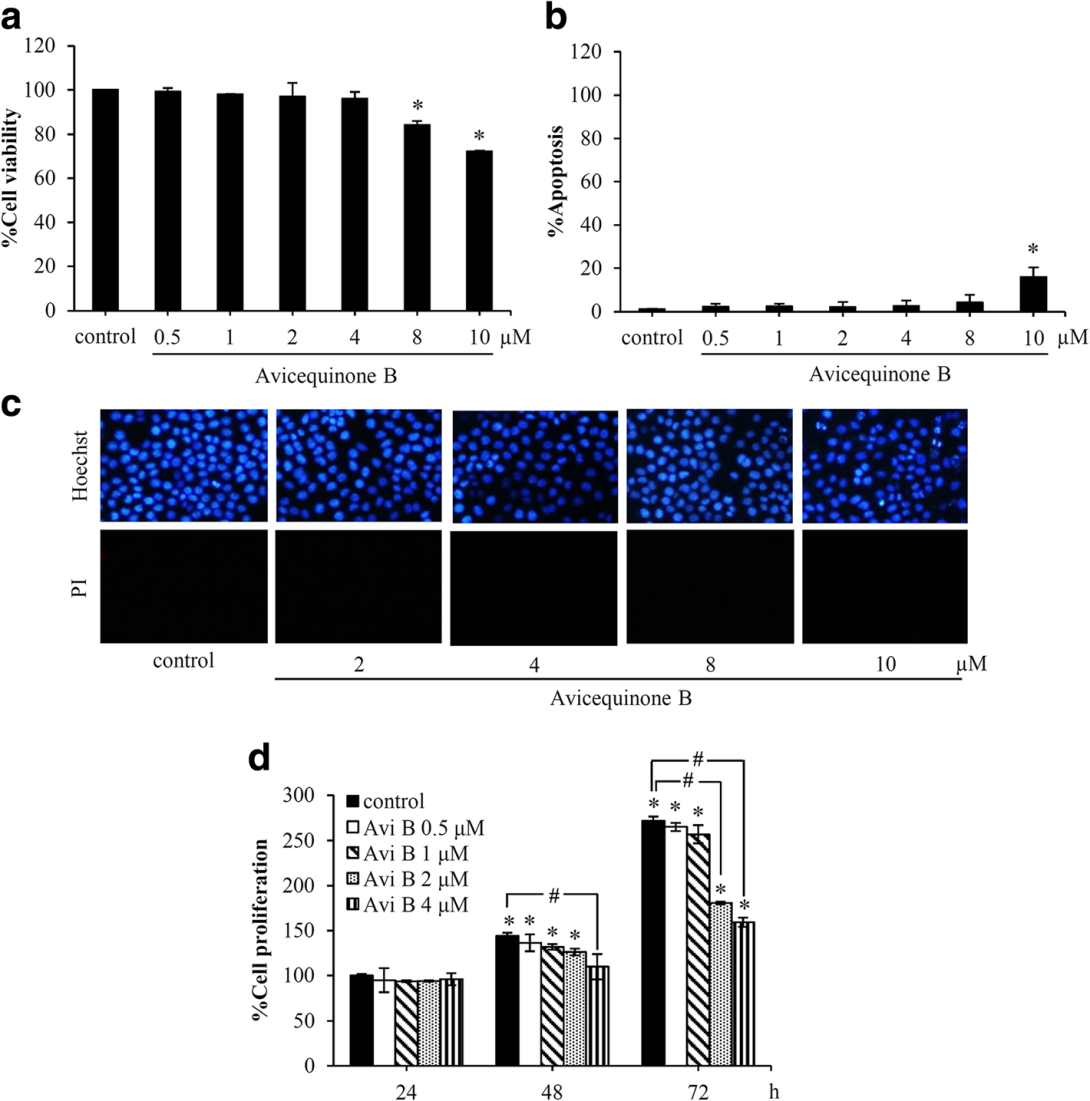
Cytotoxicity of avicequinone B in human lung cancer cells. **a** MTT assay revealed the significant reduction of cell viability in lung cancer H460 cells after treatment with 8–10 μM of avicequinone B for 24 h. **b** Avicequinone B at 10 μM induced apoptosis cell death in human lung cancer cells. **c** Co-staining with Hoechst33342/PI demonstrated that no necrosis cell death was detected in H460 cells at all treatment of avicequinone B. **d** The suppression on proliferation in adherent lung cancer cells was significantly notified in H460 cells incubated with 2–4 μM of avicequinone B for 72 h. Data represent the means ± SD (*n* = 3). *, ^*#*^ *p* ≤ 0.05 versus untreated control cells

To investigate the effect of avicequinone B on anoikis, the cytotoxicity of the compound in lung cancer H460 cells was firstly elucidated. Cell viability was examined by MTT assay after treatment of the cells with avicequinone B at 0–10 μM for 24 h. Cytotoxic profile of avicequinone B was shown in fig. 2. In detail, the significant reduction of %cell viability was observed in the cells treated with 8–10 μM of avicequinone B (Fig. 2a). Figure 2b indicates the increase of apoptosis cell death in H460 cells after treatment with 10 μM of avicequinone B. There was no observation of necrosis cells stained with red fluorescence of PI in all treatment of avicequinone B (Fig. 2c). These results demonstrated that non-toxic concentrations of avicequinone B in human lung cancer H460 cells were between 0.5 to 4 μM.

The inhibitory effect of avicequinone B on proliferation in human lung cancer cells was further evaluated. Figure 2d indicates that treatment with 2–4 μM of avicequinone B for 72 h significantly suppressed %cell proliferation in lung cancer H460 cells compared with non-treated control cells. Notably, the anti-proliferative activity of avicequinone B (4 μM) was early observed after 48 h of incubation time.

### Anoikis sensitizing effect of avicequinone B in H460 lung cancer cells

**Fig. 3:**
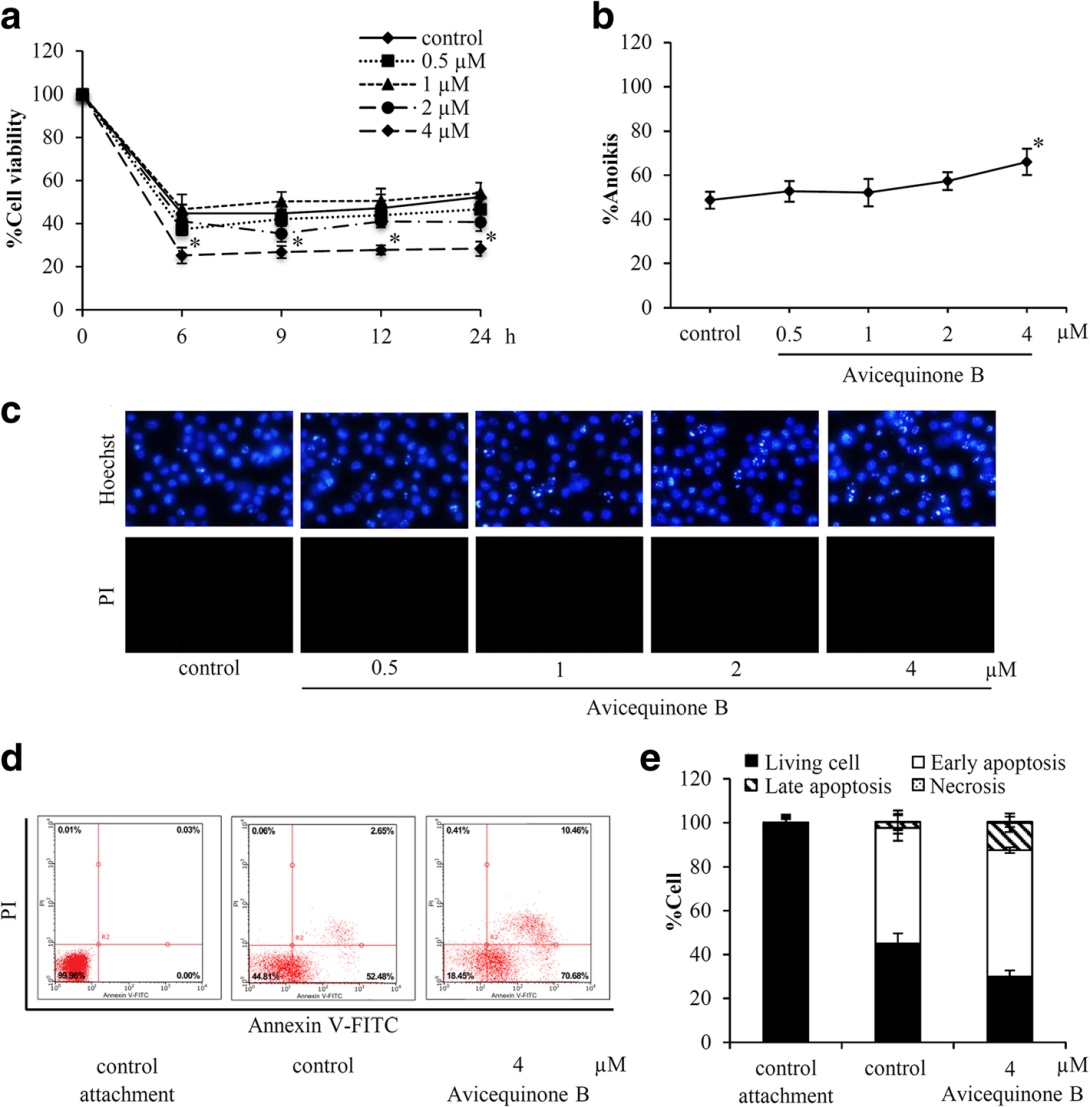
Avicequinone B sensitized anoikis in human lung cancer cells. **a** The reduction of viability was early observed in lung cancer H460 cells after culture under detachment condition for 6 h. **b** Avicequinone B at 4 μM significantly induced anoikis in H460 cells that were culture under detachment condition for 24 h. **c** Bright blue fluorescence of Hoechst33342 indicated DNA condense and apoptosis body was indicated in H460 cells treated with 4 μM of avicequinone B. **d** Detachment-induced apoptosis was evidenced with histograms obtained from flow cytometry analysis of H460 cells at anchorage culture (control attachment), detachment without treatment for 24 h (control) and detachment with 4 μM of avicequinone B for 24 h. **e** The detection early and late apoptosis without necrosis cells was remarkably increased after incubation of non-adherent H460 cells with avicequinone B at 4 μM*.* *p ≤ 0.05 versus untreated control cells at the same time point

Detachment-induced cell death was assessed in human lung cancer cells through the culture of H460 cells as single-cell suspension in non-adhesive poly-HEMA coated-plates. After 0–24 h of incubation with non-toxic concentrations (0–4 μM) of avicequinone B, the survival of the cells was determined by XTT assay. The reduction of cell survival was detected in the control cells as early as 6 h after detachment (Fig. 3a). For treated cells, avicequinone B at 4 μM significantly diminished viability of H460 cells compared with non-treated control cells at the same time point. Co-staining with Hoechst33342 and PI confirmed the anoikis sensitizing effect of avicequinone B. Induction of apoptosis without presenting of necrosis was illustrated in avicequinone B-treated H460 cells (Fig. 3c). Figure 3b shows the significant augmentation of anoikis in H460 cells after incubation with 4 μM of avicequinone B for 24 h.

Flow cytometry analysis also indicated the presence of anoikis in human lung cancer cells. Annexin V-FITC which interacts with phosphatidylserine on the cell membrane of apoptosis cells [28] was dramatically detected in H460 cells cultured in ultra-low attachment plate for 24 h (Fig. 3d). The higher number of early (Annexin V-FITC positive) and late (Annexin V-FITC positive and PI positive) apoptosis were obviously notified in detached H460 cells incubated with 4 μM of avicequinone B compared with the non-adherent control cells (Fig. 3e). These results evidenced the anoikis sensitizing activity of avicequinone B in human lung cancer cells.

### Avicequinone B suppresses cancer cell growth in anchorage-independent condition

**Fig. 4:**
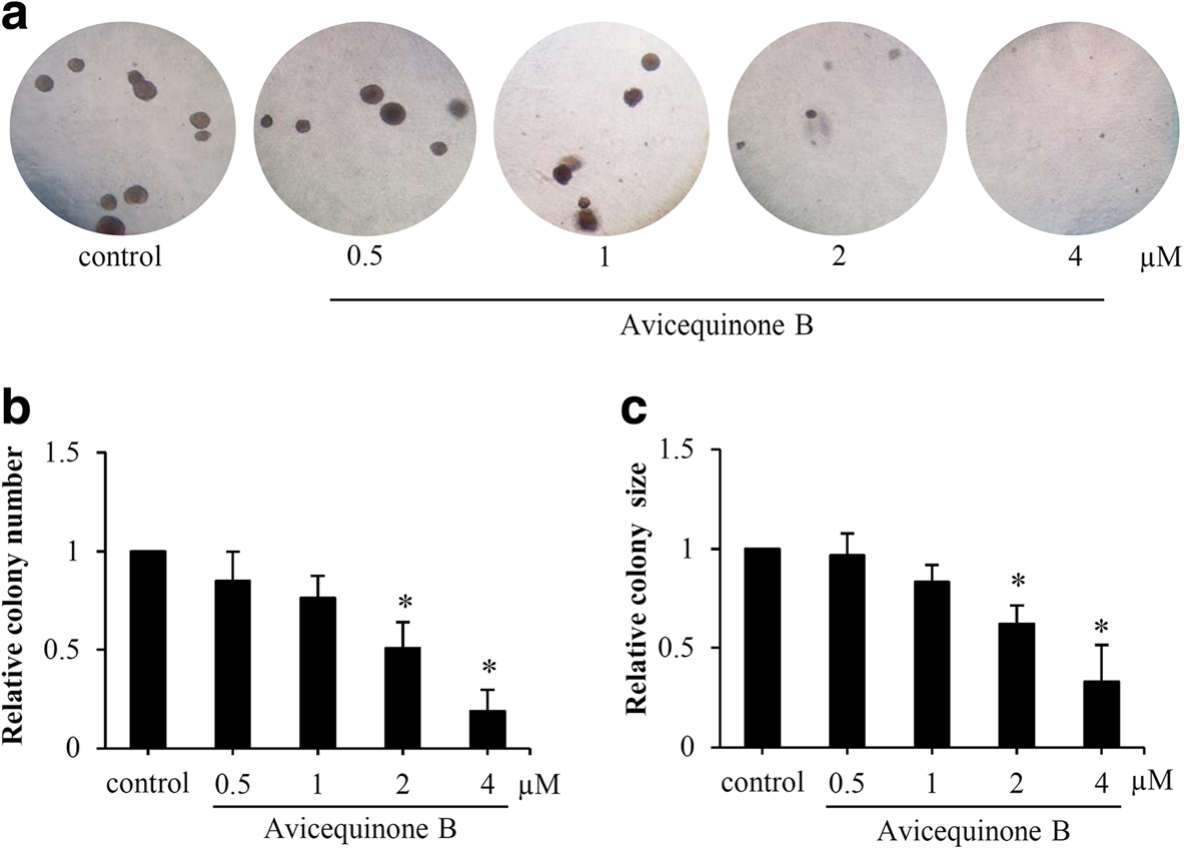
Effect of avicequinone B on anchorage-independent growth. **a** Colony formation of single-cell H460 cells was evaluated under a microscope after culture in soft agar for 14 days. **b** Relative colony number and **c** relative colony size were gradually decreased incubation of single-cell H460 with 1–4 μM of avicequinone B. Data represent the means ± SD *(n* = 3*)*. *p ≤ 0.05 versus untreated control cells

The effect of avicequinone B on capability to growth and survive under detachment condition was further evaluated in soft-agar assay. Human lung cancer H460 cells were anchorage-independently grown in 0.33% agarose gel supplemented with culture medium in presence or absence of avicequinone B (0.5–4 μM). Figure 4a presents the colony formation initiating from a single cell of H460 after 14 days of culture under detachment condition. The reduction of both relative colony number and size was significantly observed in H460 cells treated with 2–4 μM of avicequinone B (Fig. 4b and c). Intriguingly, avicequinone B at 4 μM obviously suppressed proliferation in lung cancer cells at both anchorage-dependent (Fig. 2d) and -independent condition.

### Avicequinone B decreases anti-apoptotic proteins in non-anchorage lung cancer cells

**Fig. 5:**
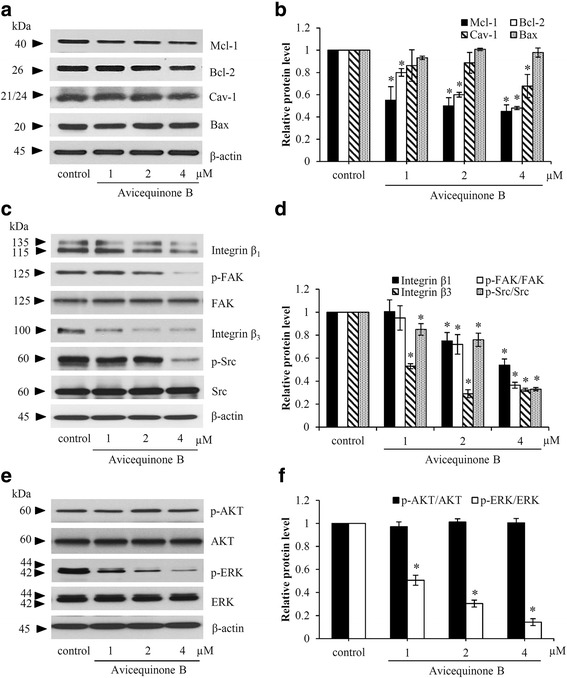
Avicequinone B down-regulated anti-apoptosis proteins and pro-survival signaling. **a** Western blot analysis revealed the reduction of anti-apoptosis proteins, Mcl-1 and Bcl-2 in H460 cells treated with avicequinone B (1–4 μM) under detachment condition for 12 h. **b** The decrease of caveolin-1 (Cav-1) was significantly notified in non-adherent H460 cells incubated with avicequinone B at 4 μM. **c** Low expression level of integrin β1 and β3 were demonstrated in avicequinone B-treated lung cancer cells. **d** Downstream pro-survival signaling of integrin including p-FAK (Y 397) associating with p-Src (Try 416) were also down-regulated after incubation of detached H460 cells with avicequinone B (1–4 μM). **e** The reduction of p-ERK (Thr 981) was significantly notified while **f** there was no alteration of p-AKT (Ser 473)/AKT in avicequinone-treated lung cancer cells. Data represent the means ± SD *(n* = 3*)*. *p ≤ 0.05 versus untreated control cells

Anoikis is apoptosis cell death induced by detachment condition, the alteration of apoptosis-regulating proteins including Bcl-2, Mcl-1, Bax and caveloin-1 was examined in H460 cells treated with avicequinone B. In order to escape from anoikis, human lung cancer H460 cells can sustain the level of anti-apoptosis proteins during non-adherent circumstance [29]. Avicequinone B at 4 μM significantly reduced the level of caveolin-1 in lung cancer cells detached for 12 h (Fig. 5a). Moreover, treatment with 2–4 μM of avicequinone B significantly declined anti-apoptotic Bcl-2 family proteins, Bcl-2 and Mcl-1 (Fig. 5b). It is worth noting that the expression of Bax, a pro-apoptosis protein was not altered in response to the treatment of avicequinone B (1–4 μM) for 12 h (Fig. 5a and b).

### Down-regulation of integrin mediated-survival signal by avicequinone B

In order to proliferate under non-adherent condition, anoikis resistant cancer cells substantially activate pro-survival pathways [9]. Thus, the alteration on integrin mediated-survival signal was investigated in H460 cells cultured with avicequinone B. Western blot analysis obviously revealed the down-regulation of integrin β1 and β3 in human lung cancer cells treated with 4 μM of avicequinone B under detachment condition (Fig. 5c). Interestingly, the reduction of integrin β3 was also notified at low concentrations (1–2 μM) of avicequinone B. Figure 5d indicates the reduction of p-FAK and p-Src, down-stream signaling molecules of integrins in H460 cells treated with avicequinone B. The alteration on level of AKT and ERK protein, pro-survival molecules activated by p-Src was further investigated [30]. As presented in fig. 5e and f, the diminution of activated ERK (p-ERK) was notified in detached H460 cells incubated with avicequinone B (1–4 μM). Meanwhile there were no significant alteration of AKT and p-AKT expression in avicequinone B treated-H460 cells compared with non-treated control. This inhibitory effect on integrin/FAK/Src survival pathway corresponded with sensitization to anoikis (Fig. 3) and low colony formation (Fig. 4) in human lung cancer cells exposed with avicequinone B.

### Avicequinone B sensitizes anoikis and suppresses anchorage-independent growth in various lung cancer cells

**Fig. 6:**
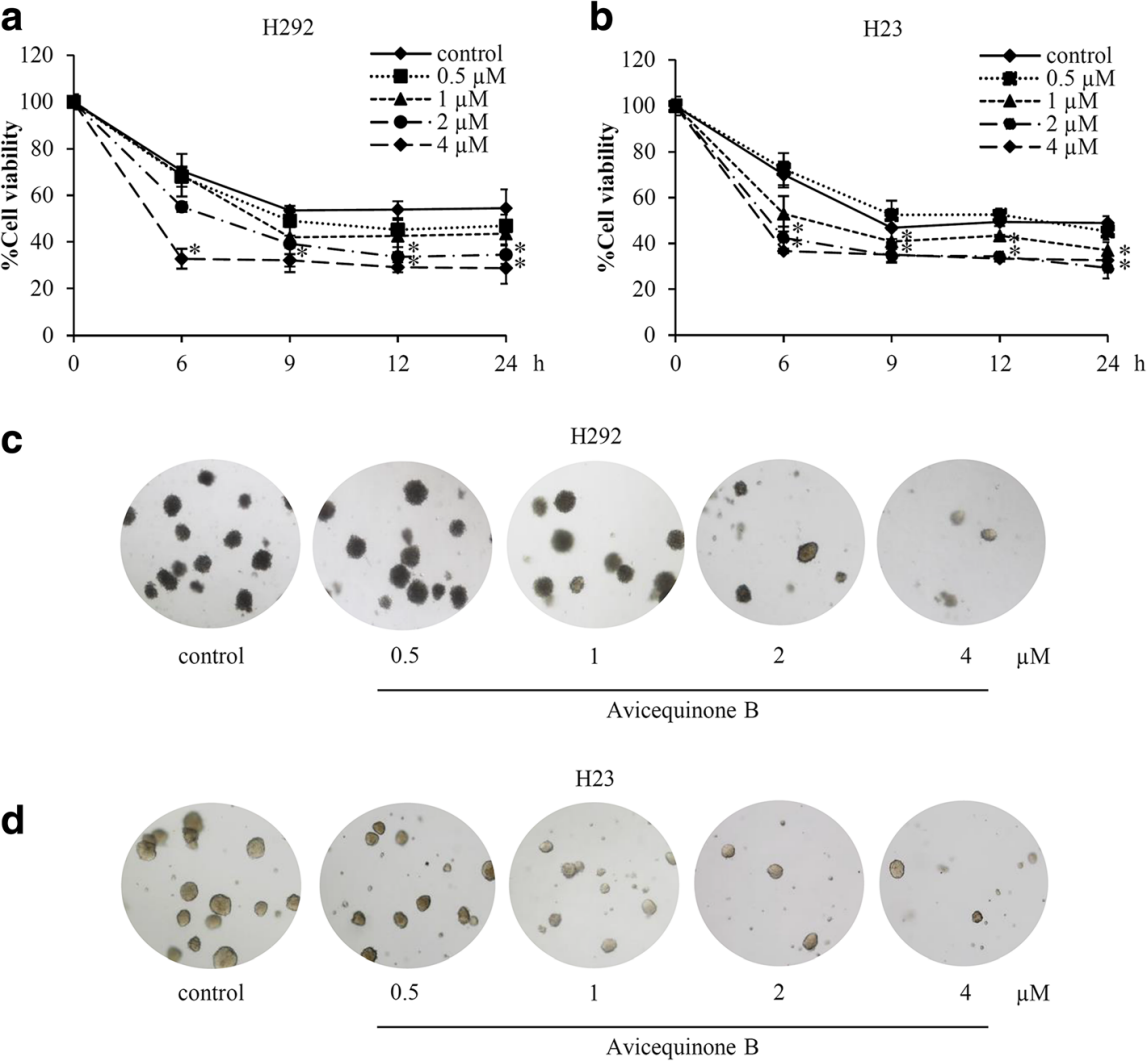
Avicequinone B restrained survival under detachment condition in various lung cancer cells. The lower viability of non-adherent **a** H292 and **b** H23 lung cancer cells was significantly notified in the cells incubated with avicequinone B at 2–4 μM compared with non-treated control. Anchorage-independent growth assay revealed the reduction of both number and size of forming colonies in lung cancer **c** H292 and **d** H23 cells after culture with 2–4 μM of avicequinone B for 14 days. Data represent the means ± SD *(n* = 3*)*. *p ≤ 0.05 versus untreated control cells at the same time point

In order to confirm anoikis sensitizing effect of avicequinone B, the detachment-induced cell death was performed in human lung cancer H292 and H23 cells. Figure 6a and b indicate that avicequinone B at non-toxic concentrations (2–4 μM; data not shown) significantly reduced viability in non-adherent H292 and H23 cells, respectively. Inspiringly, the sensitizing activity of avicequinon B on detachment-induced cell death was distinctly observed at lower dose (2 μM) in both H292 and H23 lung cancer cells compared with H460 cells which only responded to 4 μM of avicequinon B (Fig. 3a). Soft agar assay demonstrated the diminution of both colony number and size in H292 (Fig. 6c) and H23 (Fig. 6d) treated with 2–4 μM of avicequinone B. These data strengthened the anti-metastasis activity of avicequinone B on induction of anoikis and inhibition on survival under detachment condition in human lung cancer cells.

## Discussion

The capability to growth and escape from cell death under detachment condition has been found in non-adherent tumor cells circulating in blood and lymphatic system [31]. In order to resist to detachment-induced cell death, cancer cells acquire the high expression of anti-apoptosis proteins and anoikis resistant mechanisms [6, 31, 32]. Previously, caveolin-1 was shown to inhibit anoikis through the preservation of anti-apoptosis Mcl-1 protein in detached lung cancer cells [25]. Caveolin-1 was shown to be declined in response to the detachment of cells from ECM and the sustained level of the protein confers anoikis resistance [33]. Furthermore, the augmentation of Bcl-2 and diminution of Bax also manipulate anoikis in cancer cells [34]. In this study, anoikis sensitizing effect of avicequinone B in human lung cancer cells involved with the down-regulation of caveolin-1 together with the reduction of Mcl-1 and Bcl-2 (Fig. 5). Although the decrease of Mcl-1 and Bcl-2 was notified after treatment with avicequinone B at 1–4 μM, the significantly reduction of caveolin-1 and induction of anoikis were only observed in human lung cancer cells incubated with avicequinone B at 4 μM. These results support the critical role of caveolin-1 on modulation of anoikis resistance in human lung cancer cells [16, 17].

Integrins are transmembrane molecules that regulate not only cell adhesion but also various cellular signaling pathways [8]. The interaction between integrins and protein components in ECM or specific ligands activate downstream FAK at focal adhesion complex consequence with phosphorylation of pro-survival proteins. In spite of loss of anchorage with ECM, highly metastatic cancer cells are able to generate integrin survival signals resulting in resistance to anoikis [10, 12, 35]. The up-regulation of integrin β1 and β3 influences with various aggressive behaviors in cancer cells [36, 37]. Recent study revealed that the diminution of β1 and β3 integrins suppresses survival and growth under detachment condition in human lung cancer cells [38]. Herein, suppression on integrin survival pathway in lung cancer cells induced by avicequinone B was elucidated. There was the reduction of integrin β1 and β3 level consequence with the restrain of downstream signaling molecules, p-FAK and p-Src in non-adherent lung cancer cells treated with avicequinone B.

The up-regulation of anti-apoptotic proteins and pro-survival signal has been revealed in lung cancer cells with anoikis resistant phenotype [39]. Down-regulation of caveolin-1, Mcl-1 and Bcl-2 as well as suppression on AKT and ERK activation successfully initiate detachment-induced cell death in anoikis-resistant lung cancer cells [40, 41]. The suppression on integrin/FAK/Src pathway consequence with the reduction of pERK/ERK and diminution of anti-apoptosis Bcl-2 family proteins evidence the sensitizing effect of avicequinone B in anoikis resistant lung cancer cells.

Phosphorylation on FAK and Src leading to formation FAK-Src complex which modulates metastasis features such as migration and anchorage-independent growth [42]. Therefore, the suppression on integrin/FAK/Src signaling has been recognized as targeted pathway for treatment metastasis cancer [43]. Inhibition on migration and invasion of naphthoquinone compound has been demonstrated in cancer cells [22]. Taken together with the anoikis sensitizing activity of avicequinone B obtained from this study, these data strengthened the possibility to develop naphthoquinones and their derivatives as novel anti-metastasis drugs.

## Conclusions

**Fig. 7:**
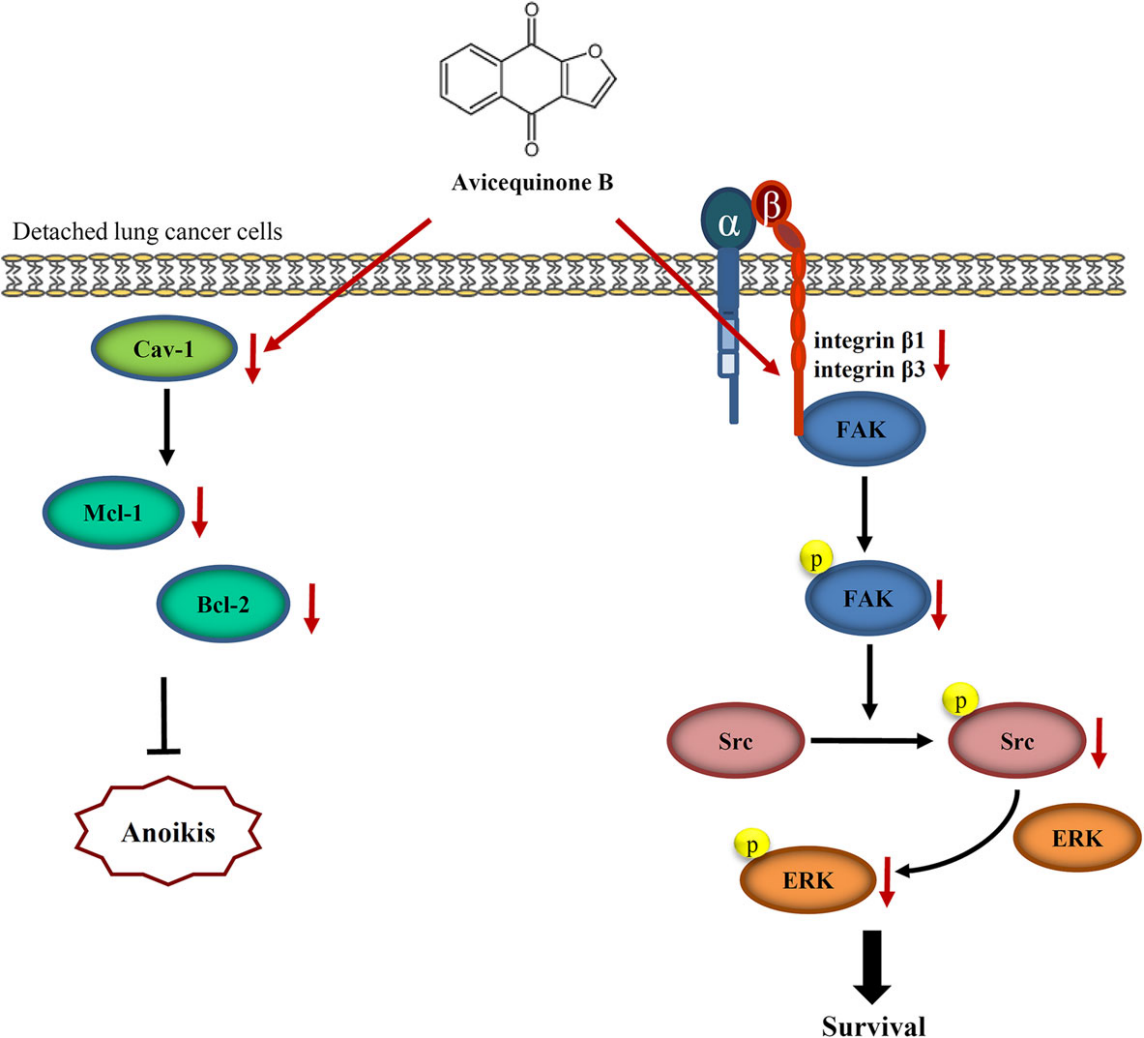
Proposed mechanism of avicequinone B-induced anoikis in lung cancer cells. Avicequinone B mediated anoikis through the reduction of anti-apoptosis proteins caveloin-1, Mcl-1 and Bcl-2 as well as down-regulation of integrin/FAK/Src pro-survival pathway

In conclusion, our study provided the evidence indicating that avicequinone B suppressed survival and induced anoikis in human lung cancer cells under detachment condition through inhibition on integrin/FAK/Src signaling and down-regulation of anti-apoptosis protein including cavelolin-1, Mcl-1 and Bcl-2 (Fig. 7). These data support the further development of avicequinone B as an effective treatment to overcoming cancer metastasis.

## Abbreviations

AKT: Phospho kinase B
Bax: Bcl-2-associated X protein
BCA: Bicinchoninic acid
Bcl-2: B-cell lymphoma 2
Cav-1: Caveolin-1
DBU: 1,8-diazabicyclo[5.4.0]undec-7-ene
DMSO: Dimethysulfoxide
ECM: Extracellular matrix
ERK: Extracellular signal–regulated kinase
FAK: Focal adhesion kinase
FBS: Fetal bovine serum
HRP: Horseradish peroxidase
Mcl-1: Myeloid cell leukemia 1
MTT: 3-(4,5-Dimethylthiazol-2-yl)-2,5-diphenyltetrazolium bromide
OD: Optical density
p-AKT: Phosphorylated phospho kinase B
PBS: Phosphate buffer saline
p-ERK: Phosphorylated extracellular signal–regulated kinase
p-FAK: Phosphorylated focal adhesion kinase
PI: Propidium iodide
PI3K: Phosphatidylinositol-4,5-bisphosphate 3-kinase
p-Src: Phosphorylated proto-oncogene tyrosine-protein kinase
RPMI: Roswell Park Memorial Institute
SDS-PAGE: Sodium dodecyl sulfate polyacrylamide gel electrophoresis
Src: Proto-oncogene tyrosine-protein kinase
THF: Tetrahydrofuran
XTT: 2,3-b-(2-methoxy-4-nitro-5-sulfophenyl)-2H-tetrazolium-5-carboxanilide salt
